# Plasma level of cyclophilin A is increased in patients with type 2 diabetes mellitus and suggests presence of vascular disease

**DOI:** 10.1186/1475-2840-13-38

**Published:** 2014-02-07

**Authors:** Surya Ramachandran, Anila Venugopal, V Raman Kutty, Vinitha A, Divya G, V Chitrasree, Ajit Mullassari, N S Pratapchandran, K R Santosh, M Radhakrishna Pillai, C C Kartha

**Affiliations:** 1 Cardiovascular Disease Biology, Rajiv Gandhi Centre for Biotechnology, Thiruvananthapuram, India; 2 Achutha Menon Centre, SCTIMST, Thiruvananthapuram, India; 3 Madras Medical Mission, Chennai, India; 4 Indian Institute of Diabetes, Thiruvananthapuram, India; 5 PRS Hospital, Thiruvananthapuram, India; 6 Cancer Research Program, Rajiv Gandhi Centre for Biotechnology, Thiruvananthapuram, India

**Keywords:** Hyperglycemia, Type 2 diabetes mellitus, Monocytes, Cyclophilin A, Vascular disease

## Abstract

**Aims/hypothesis:**

Cyclophilin A, an immunophilin is secreted from human monocytes activated by high glucose. Given its role as an inflammatory mediator of vascular tissue damage associated with inflammation and oxidative stress, we examined plasma levels of cyclophilin A in normal healthy volunteers and patients with type 2 diabetes (DM), with or without coronary artery disease (CAD).

**Methods:**

Study subjects comprised of 212 patients with DM and CAD,101 patients with diabetes, 122 patients with CAD and 121 normal healthy volunteers. Diabetes was assessed by HbA1c levels while coronary artery disease was established by a positive treadmill test and/or coronary angiography. Plasma cyclophilin A was measured using a cyclophilin A ELISA Kit. Relationship of plasma cyclophilin A levels with blood markers of type 2 diabetes, blood lipid levels and medication for diabetes and coronary artery disease were also explored.

**Results:**

Plasma Cyclophilin levels were higher in diabetes patients with or without CAD compared to normal subjects (P < 0.001). Age, fasting blood sugar levels and HbA1C levels were positively associated with increased plasma cyclophilin. Patients using metformin had reduced levels of plasma cyclophilin (p < 0.001).Serum levels of total cholesterol, LDL cholesterol and triglycerides had no significant association with plasma cyclophilin levels. In patients with increased serum CRP levels, plasma cyclophilin A was also elevated (p = 0.016). Prevalence odds for DM, DM + CAD and CAD are higher in those with high cyclophilin values, compared to those with lower values, after adjusting for age and sex, indicating strong association of high cyclophilin values with diabetes and vascular disease.

**Conclusions/interpretations:**

Our study demonstrates that patients with type 2 diabetes have higher circulating levels of cyclophilin A than the normal population. Plasma cyclophilin levels were increased in patients with diabetes and coronary artery disease suggesting a role of this protein in accelerating vascular disease in type 2 diabetes. Considering the evidence that Cyclophilin A is an inflammatory mediator in atherogenesis, the mechanistic role of cyclophilin A in diabetic vascular disease progression deserves detailed investigation.

## Introduction

Cyclophilin A is part of various intracellular functions, such as intracellular signaling, protein trafficking, and regulating activity of other proteins [1]. Cyclophilin A, is also well recognized as a secreted growth factor that is induced by oxidative stress [2] functioning as a mediator of tissue damage associated with inflammation and oxidative stress [3]. The secretory nature of this protein and its presence in plasma of patients with DM and CAD underlines its potential as a marker of disease. We earlier reported proteomic changes occurring in circulating monocytes on activation with high glucose. We also demonstrated secretion of cyclophilin A from monocytes under hyperglycemic conditions. Preliminary studies on a small number of patients with type 2 diabetes mellitus and coronary artery disease revealed increased plasma levels of cyclophilin A compared to normal healthy individuals [4].

We report here the results of an enzyme linked immunosorbent assay based study in a larger study population comprising 556 subjects, consisting of patients with type 2 diabetes mellitus with or without coronary artery disease, patients with only coronary artery disease and normal healthy volunteers. Levels of plasma cyclophilin A in these study groups was determined and correlated with biochemical markers of diabetes and blood lipid profile.

## Methods

### Study participants

The study was done on patients with type 2 diabetes with or without macrovascular complications such as coronary artery disease. Patients were recruited from three multispecialty hospital centers in Southern India. All the protocols were submitted to and approved by the Human Ethics Committee of Rajiv Gandhi Centre for Biotechnology and participating hospitals (Indian Institute of Diabetes, PRS Hospital and Madras Medical Mission). A written informed consent was obtained from all study subjects. All patients subsequently underwent a standardized clinical and laboratory evaluation.

The study subjects (n = 556) were divided into five groups: (i) Patients with type 2 diabetes mellitus (DM; n = 101), (ii) diabetes patients with coronary artery disease (DM + CAD) diagnosed within 5 years of detection of type 2 diabetes (n = 103), (iii) patients with DM who had CAD diagnosed five years after detection of diabetes (n = 109), (iv) patients with CAD (n = 122) and without diabetes and (v) normal healthy volunteers (n = 121).

Patients with hypertension, cardiac diseases other than coronary artery disease, peripheral vascular disease, thrombotic stroke, nephropathy, retinopathy, inflammatory disease of any cause, subjects with other systemic and metabolic diseases, asthma, malignancy, liver diseases, kidney diseases and pregnant women were excluded from the study. Diabetes was assessed by recording HbA1c and/or fasting blood sugar (FBS) levels while CAD was diagnosed by a positive treadmill test and/or coronary angiography.

### Enzyme linked assay for measurement of cyclophilin A in plasma

Blood samples were collected after overnight fasting. Blood was collected into EDTA containing tubes and centrifuged for 7 minutes at 2500 g within 30 minutes of collection. The separated plasma was transferred to a fresh tube and used directly for the immunoassay as per the supplier’s protocol. Cyclophilin A levels in plasma were determined with a sandwich immunoassay kit (Uscn Life Science Inc, Product No. SEA979Hu). The linearity of the kit was assayed by testing samples spiked with a known concentration of cyclophilin A and their serial dilutions. Spiking with known concentrations of the protein guaranteed a recovery range of 83–102%. No significant cross-reactivity or interference with any other proteins was observed. All samples were analyzed in duplicate. To maintain assay precision, samples with a CV > 12% were excluded.

C-Reactive Protein (CRP) was measured with a quantikine sandwich Human CRP Immunoassay (R & D Systems, Product No: DCRP00). A 93-100% recovery range was observed when samples were spiked with 1:2 to 1:16 concentrations of CRP.

Medication history was recorded for 270 subjects of the 556 subjects. Medications were grouped into (i) antiplatelet aggregating agents (Ecospirin, Clopidogrel, Cilostazol), (ii) Antihypertensive agents (Calcium channel blockers, ACE inhibitors, vasodilators), (iii) Statins (atorvastatin, Lovastatin) and (iv) Metformin derivatives (glucophage, glycimet).

### Statistical analysis

The distribution of all variables in the five groups was studied by describing the mean, median, range and standard deviation. Predictor variables such as age, HbA1C, fasting blood sugar (FBS) were grouped into two based on median values; cyclophilin A levels in the two groups compared using student t test. The same procedure was used for comparing cyclophilin levels in groups with different levels of CRP, and in patients using metformin or not. Cyclophilin values in the five groups were compared with ANOVA (F = 54.75, p < 0.001), followed by multiple comparisons using pairwise t tests with pooled variance by the Holms’ method. We did a multinomial logistic regression analysis for estimating the prevalence odds ratios for presence of disease, with the normal subjects as the reference. The odds for prevalence of the other four conditions, i.e., diabetes and no heart disease (DM), heart disease and no diabetes (CAD), diabetes and heart disease of up to 5 years duration (DM + CAD 5Y), and diabetes and heart disease beyond 5 years but below 10 years (DM + CAD 10Y), among those subjects with high cyclophilin levels were compared with prevalence odds in those with low cyclophilin values, adjusted for age and sex. All statistical tests were done with an estimated power =0.80. All statistical tests were done in R (R Core Team , 2013, R: A language and environment for statistical computing. R Foundation for Statistical Computing, Vienna, Austria.); graphs were also created in R.

## Results

### Baseline clinical parameters of patients with diabetes, coronary artery disease and controls

A total of 313 diabetes patients; 212 with CAD and 101 without CAD (210 men and 103 women), 122 patients with only coronary artery disease (102 men and 20 women) and 121 normal healthy volunteers (66 men and 55 women) without any evidence of systemic disease participated in the study. The distribution of study subjects and their baseline clinical characteristics are described in Table 1. The mean values and standard deviation of each clinical parameter is given in Table 2. Mean HbA1c values were highest in the diabetes mellitus (DM) group (mean: 8.3; Std dev: 1.3) followed by the group of patients with diabetes and coronary artery disease (DM + CAD10y) (mean: 8.6, Std dev: 2.0). Triglyceride levels were higher in patients with coronary artery disease irrespective of whether they had diabetes compared to normal subjects and patients with only diabetes.

**Table 1:** Frequencies of baseline clinical parameters of the study groups

	**Normal N (%)**	**DM N (%)**	**CAD N (%)**	**DM+CAD (0-5y) N (%)**	**DM**+**CAD (5-10y) N (%)**
**Age**					
**>** ***45***	47 (38.8)	18 (18)	13 (10.6)	6 (5.9)	3 (2.8)
**<** ***45***	74 (61.1)	83 (83)	109 (85.3)	97 (95.1)	106 (97.2)
**Gender**					
*** Male***	66 (54.5)	49 (49)	102 (83.6)	83 (81.4)	78 (71.6)
*** Female***	55 (48.4)	52 (52)	20 (16.3)	20 (19.6)	3 (28.4)
**FBS (mg/dL)**					
*** 70*-*110***	89 (73.5)	26 (26)	43 (35.2)	25 (24.5)	21 (19.3)
**>** ***110***	32 (26.4)	75 (75)	79 (64.7)	78 (76.5)	88 (80.7)
**HbA1C (%)**					
**<** ***5.7***	88 (72.7)	4 (4)	50 (40.9)	1 (1)	3 (2.8)
***5.7*-*6.4***	28 (23.1)	6 (6)	40 (32.70	19 (18.6)	5 (4.6)
**>** ***6.5***	5 (4.1)	91 (91)	32 (26.2)	83 (81.4)	101 (92.7)
**Total cholesterol (mg/dL)**					
**<** ***200***	64 (52.8)	58 (58)	90 (73.7)	78 (76.5)	80 (73.4)
***200*-*240***	37 (30.5)	36 (36)	15 (72.2)	12 (11.8)	13 (11.9)
**>** ***240***	20 (16.5)	7 (7)	17 ( (13.9)	13 (12.7)	16 (14.7)
**LDL (mg/dL)**					
**<** ***100***	91 (75.2)	33 (33)	78 (63.9)	68 (66.7)	64 (58.7)
**>** ***100***	30 (24.7)	68 (68)	44 (36.1)	35 (34.3)	45 (41.3)
**HDL (mg/dL)**					
**<** ***60***	113 (43.3)	90 (90)	117 (95.9)	100 (98)	104 (95.4)
**>** ***60***	8 (6.6)	11 (11)	5 (4.1)	3 (2.9)	5 (4.6)
**Triglycerides (mg/dL)**					
**<** ***150***	113 (43.3)	90 (90)	78 (63.9)	64 (62.7)	77 (70.6)
**>** ***150***	8 (6.6)	11 (11)	44 (36.1)	39 (38.2)	32 (29.4)

**Table 2:** Mean values of clinical parameters in the study groups

	**N**		**DM**		**CAD**		**DM+CAD 5y**		**DM+CAD 10y**	
	**M**	**SD**	**M**	**SD**	**M**	**SD**	**M**	**SD**	**M**	**SD**
**Age**	41.6	11.2	55.2	10.0	58.9	11.2	58.8	9.0	62.4	8.4
**FBS**	106.6	25.8	149.8	54.6	124.8	31.4	145.6	52.8	162	58.1
**HbA1c**	5.3	0.5	8.3	1.3	6.0	1.1	7.7	1.6	8.6	2.0
**Total cholesterol**	199	42	192.9	34.5	167.7	56.2	172.1	60.6	170.5	56.0
**LDL**	92.1	23.9	110.6	22.5	102	36.6	96.9	40.2	105.5	39.3
**HDL**	43.5	8.5	47.4	9.2	42.4	12.0	36.1	10.1	38.6	11.7
**Triglycerides**	107.4	29.6	103.2	33.2	136.6	49.0	152.9	81.5	134.4	58.4
**Cyclophilin A**	13.2	3.8	16.1	4.0	20.0	8.0	18.7	9.4	19.3	8.2

Mean values of clinical parameters in the study groups. *N* = Normal, *DM *= Diabetes mellitus, *CAD *= Coronary artery disease, *DM*+*CAD*5y = diabetes patients with coronary artery disease (DM+CAD) diagnosed within 5 years of detection of type 2 diabetes, DM+CAD10y = patients with DM who had CAD diagnosed five years after detection of diabetes; M = Median values and *SD *= Standard deviation.

### Levels of plasma cyclophilin A and association with disease

**Figure 1:**
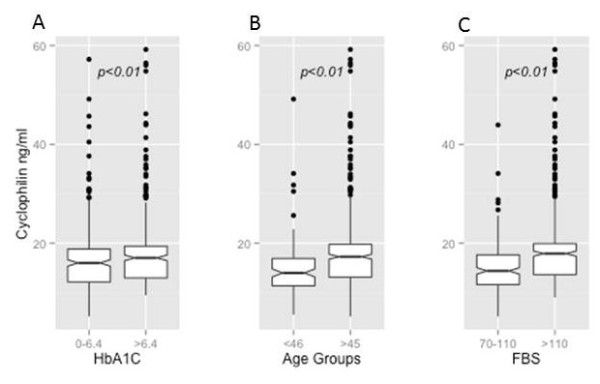
**Box and whisker plot of association between (A) HbA1C (B) Age and (C) Fasting blood sugar (FBS) and plasma cyclophilin levels.** HbAlc was divided into <6.4 and 6.4 and above age was divided into <46 and >45 and FBS between 70-110 mg/dL and >110 mg/dL.

**Figure 2:**
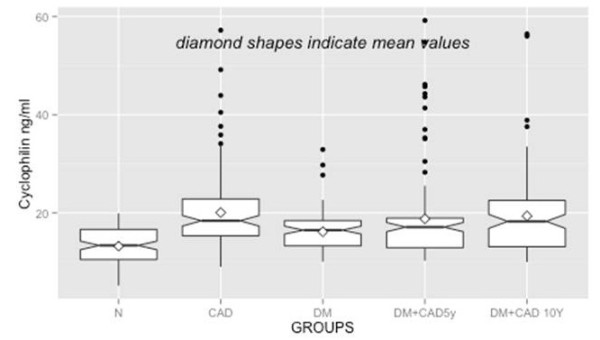
**Box and whisker plot of distribution of cyclophilin levels in plasma of the various study groups.** Nt = Normal control; CAD = Coronary artery disease; DM = Diabetes Mellitus, DM+CAD5y = diabetes patient diagnosed with CAD in 5 years and DM+CAD1Oy = diabetes patients diagnosed with CAD within 10 years. P values were <0.001 for all groups.

Plasma cyclophilin in the 556 subjects ranged from 5.9 ng/ml to 59.2 ng/ml) with a median value of 16.7 ng/ml. Subjects were divided into two groups: subjects with plasma cyclophilin <16.7 ng/ml and those above 16.7 ng/ml. There was no significant difference in plasma cyclophilin levels between males and females (p = 0.72). We observed that only age (t = 3.93; p < 0.01), FBS (t = 6.19; p < 0.01) and HbA1C (t = 2.34; p = 0.019) were associated with changes in plasma cyclophilin A levels (Figure 1). Other variables such as serum levels of total cholesterol, LDL cholesterol and triglycerides had no significant association with plasma cyclophilin levels. Figure 2 describes the distribution of cyclophilin levels among the various study groups. The plasma levels of cyclophilin A were significantly higher in patients with type 2 diabetes (DM) in comparison to normal volunteers (p < 0.001). Plasma levels were of normal distribution in the control group whereas in other groups the distribution was skewed. We used ANOVA to look into the association of cyclophilin A levels in 5 groups of subjects: (i) patients with diabetes (DM) and diagnosed to have CAD within 5 years of onset of diabetes (DM + CAD5y), (ii) patients with DM and diagnosed to have CAD after 5 years and within 10 years of onset of diabetes (DM + CAD 10y), (iii) patients with only diabetes, (iv) patients with only diabetes, and (v) controls. The analysis revealed that the normal group (N) was different from all other groups, and the diabetic group (DM) was different from all other groups. The CAD group did not differ from the DM + CAD groups (both DM + CAD5y and DM + CAD10y) with respect to cyclophilin levels, but were distinctly different from normal (N) as well as diabetes patient (DM) group.

Multinomial logistic regression analysis revealed that prevalence odds for all four conditions (DM, DM + CAD 5Y, DM + CAD 10y and CAD) are higher in those with high cyclophilin values, compared to those with lower values, after adjusting for age and sex, indicating strong association of high cyclophilin values with diabetes and vascular disease. However, the prevalence odds of those with both diabetes and heart disease were lower compared to those subjects who had only heart disease; the last mentioned group had the strongest association with high cyclophilin values. The prevalence odds ratios and 95% confidence interval are given in Table 3.

**Table 3:** **Prevalence Odds Ratios** (**POR**) **and 95**% **confidence intervals for association of disease conditions with high vs low cyclophilin levels**, **adjusted for age and sex**, **using multinomial logistic regression analysis**

Groups*	**Prevalence odds ratio**	**95% CI on POR**
**DM (n=101)**	2.37	1.26-4.44
**DM+CAD 5Y (n=103)**	2.99	1.56-5.71
**DM+CAD 10Y (n=109)**	3.76	1.97-7.17
**CAD (n=122)**	5.17	2.76-9.71

*Normal subjects were the referent group. DM = subjects with diabetes only; DM+CAD 5Y = subjects with diabetes and coronary disease of < 5 years’ duration; DM+10Y = subjects with diabetes and coronary disease with duration <10 years but > 5years.

### Association of C reactive protein with cyclophilin A levels

**Figure 3:**
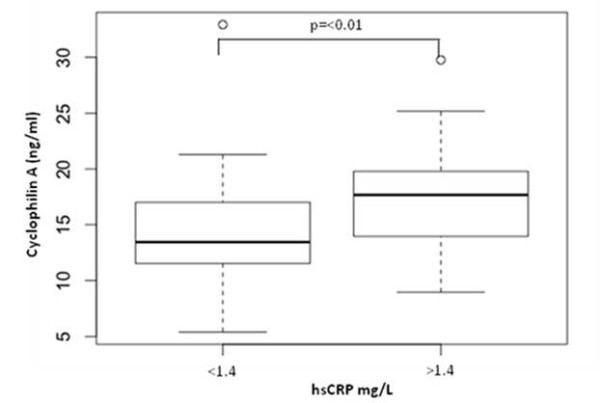
**Box and whisker plot of correlation between hsCRP levels and plasma cyclophilin levels.** hsCRP levels of patients were grouped into <1.4 mg/I (low risk) and 1.4 mg/I and above (high risk).

It is well recognized that patients with low serum CRP (<1 mg/L) are at low risk and those with higher CRP (>1 mg/L) are at moderate or high risk for future cardiovascular events [5]. We evaluated the plasma levels of cyclophilin A among patients with high and low levels of CRP. Plasma cyclophilin A levels in patients with higher serum CRP (>1.4 mg/L, n = 38) and lower serum CRP (<1.4 mg/L, n = 39) are shown as box-and-whisker plots in Figure 3. There was a positive association between higher serum CRP levels and higher plasma cyclophilin A levels (P = 0.016) compared with plasma cyclophilin levels in the low serum CRP group.

### Cyclophilin levels and medication

Cyclophilin levels were positively associated with use of metformin (Table 4). The plasma levels of cyclophilin were low in those regularly taking metformin (p < 0.001). None of the other medications were found to be associated with plasma cyclophilin A levels.

**Table 4:** **Association of medications for diabetes**, **coronary artery disease and plasma levels of cyclophilin**

Medication*	**Mean**	**Median**	**Std Dev**	**Min range**	**Max range**	**T value**	**P value**
**(i) Antiplatelets**							
*Yes*	15.31	15.58	3.2	9.03	5.54	1.52	0.12
*No*	14.67	15.12	3.6	5.54	19.93		
**(ii) Statins**							
*Yes*	15.25	15.58	3.3	9.03	22.73	1.28	0.20
*No*	14.71	15.38	3.5	5.54	19.93		
**(iii) Antihypertensive**							
*Yes*	15.14	15.54	3.3	6.92	22.73	0.84	0.40
*No*	14.78	15.4	3.5	5.54	19.93		
**(iv) Metformin**							
*Yes*	15.75	16.58	3.2	10.28	22.73	**4.00**	<**0.001**
*No*	14.1	14.33	3.5	5.54	19.92		

*Medications were grouped into (i) antiplatelet aggregating agents (Ecospirin, Clopidogrel, Cilostazol) (ii) Statins (atorvastatin, lovastatin) (iii) Antihypertensive agents (Calcium channel blockers, ACE inhibitors, vasodilators and (iv) metformin derivatives (Glucophage, glycimet).

## Discussion

We describe here the difference in circulating levels of plasma Cyclophilin A in patients with diabetes in comparison to normal individuals without any systemic disease. We used an ELISA method to quantify the cyclophilin A levels in human plasma. The method is accurate and specific for detection of the immunophilin in plasma. Cyclophilin A was considerably high in the plasma of patients with type 2 diabetes. The levels were also higher in plasma of diabetes patients who also had coronary artery disease.

### Cyclophilin as an oxidative stress induced secretory factor

Cyclophilin A has been demonstrated to be an important secreted oxidative stress induced factor [6]. Cyclophilin A is secreted from vascular smooth muscle cells (VSMCs) [7] and endothelial cells (EC) [8] in response to reactive oxygen species (ROS). Cyclophilin A also stimulates smooth muscle cell migration and proliferation, endothelial cell adhesion molecule expression and chemotaxis of inflammatory cells. Studies on cyclophilin knockout mice models have demonstrated increased atherosclerotic potential clearly indicating its role in vascular disease progression [6]. Elevated levels of extracellular cyclophilin have also been detected in the synovial fluid of patients with rheumatoid arthritis [9]. Studies have reported an interaction between extracellular cyclophilin and CD 147 expressed by macrophages which may be responsible for development of arthritis [10]. Cyclophilin A has been shown to elicit inflammatory responses and thus contribute to recruitment of circulating blood cells during inflammatory response. Secreted cyclophilin A also contributes to maintaining blood brain barrier integrity and reducing tissue damage following brain injury. It reduces brain permeability by activating survival and growth pathways. Secreted cyclophilin A activates endothelial cells, a major component of blood brain barrier which in turns contributes to recruitment of circulatory monocytes to aid in blood brain barrier repair [11]. Given that cyclophilin A is a potent chemotactic agent for immune cells, particularly monocytes; it is plausible that this immunophilin plays an important role in accelerating atherosclerosis in type 2 diabetes.

Apart from its secretory features, cyclophilin has also gained recognition from a methodological point of view. As diabetes can modulate heart glyceraldehyde 3-phosphate dehydrogenase (GAPDH) content expression, data can be normalized to cyclophilin expression [12]. Moreover, target gene expression may be calculated by using the expression of the cyclophilin housekeeping gene as an internal standard [13].

### Plasma cyclophilin A levels in diabetes and coronary artery disease

We had earlier reported that high glucose induces secretion of cyclophilin A from monocytes [4]. We also observed a decrease in expression of this protein in the monocytes of patients with diabetes mellitus when compared to its expression in healthy controls [4]. Hyperglycaemia in diabetes and related oxidative stress could contribute to secretion of cyclophilin A from circulating monocytes and a rise in plasma cyclophilin A levels as seen in our patients with diabetes. Plasma cyclophilin levels have been assessed earlier in patients with coronary artery disease to study the severity of CAD using an immunoassay similar to the one we employed in our present study [14]. Satoh et al. divided cyclophilin values into equal quartiles for their analysis. A positive correlation was noted between plasma cyclophilin levels and coronary stenosis. Higher quartiles of plasma cyclophilin levels were associated with the need for coronary intervention. We also observed that patients with CAD, irrespective of whether they had diabetes had significantly higher cyclophilin A levels than those without CAD. This indicates the possible role of cyclophilin A in the pathogenesis of vascular disease. We had divided the patients into two groups based on when CAD was detected in them. This was done to evaluate whether cyclophilin A levels in plasma were different in chronic patients. Interestingly cyclophilin A levels were higher in chronic diabetes with CAD group. The significance of this finding needs to be evaluated through a prospective study.

The high prevalence of diabetes with and without coronary artery disease in those with high blood cyclophilin levels revealed in multinomial logistic regression analysis indicate a strong association of high plasma cyclophilin values with diabetes and vascular disease. The lower degree of association of high plasma cyclophilin levels with diabetes compared to those patients with only coronary artery disease could perhaps be explained by the possible higher mortality and lower survival among with those with multiple disease, resulting in lower prevalence.

Our study also demonstrates that classical risk factors and markers of diabetes, such as age, fasting blood sugar (FBS) and glycated hemoglobin (HbA1c) were positively associated with plasma cyclophilin levels indicating a specific relation of plasma cyclophilin levels with hyperglycemia. Other parameters such as gender, family history of diabetes, serum levels of high and low density lipoproteins and triglycerides were not associated with increase in cyclophilin levels. Satoh et al. also found that age, diabetes and hsCRP correlate with plasma cyclophilin levels in their patients with stenotic coronary arteries [14].

### Plasma cyclophilin A is associated with C reactive protein levels

We next considered the correlation between plasma cyclophilin A and serum C reactive protein (CRP), a clinical marker of vascular inflammation [5]. CRP, an acute phase reactant has been developed as a surrogate marker of inflammatory mediators in coronary artery disease and diabetes [15]–[18]. The classical prospective study by Ridker et al. found elevated CRP to be an independent risk determinant of type 2 diabetes in healthy middle aged women [19]. CRP levels are observed to be higher in many diseases such as infections, trauma, or malignancy, as well as inflammatory, allergic, or necrotic diseases [20]. We evaluated the role of cyclophilin A among patients with high and low levels of serum CRP. We had looked into serum levels of CRP in a small limited subset of 77 individuals. Patients with low CRP (<1 mg/L) are at low risk and those with higher CRP (>1 mg/L) are at moderate or high risk for future cardiovascular events. We observed a positive correlation between plasma cyclophilin A and serum CRP levels. There was a positive association between patients with higher hsCRP levels and those with elevated plasma cyclophilin A in patients with diabetes as well as those with diabetes and CAD. Correlation (Pearson) Co-efficients of cyclophilin values were however, not significant between CRP and cyclophilin values possibly due to limited sample subset. A study by Satoh et al. [14] had suggested the use of cyclophilin A in conjunction with CRP as a predictor of risk in coronary artery disease in a study comprising of 320 patients with CAD. These findings suggest the possible utility of plasma cyclophilin A level as a marker of proinflammatory status in patients with diabetes.

### Metformin may reduce plasma cyclophilin levels

To understand the variation in cyclophilin levels in relation to use of medication for diabetes and coronary artery disease, we looked into the association of plasma cyclophilin levels with the intake of medicines prescribed for these diseases. Cardiovascular drugs such as aspirin, clopidogrel and statins did not have any effect on plasma cyclophilin levels, whereas metformin intake was associated with decreased plasma cyclophilin levels. Metformin is a standard hypoglycemic drug used in treatment of non-insulin dependent diabetes mellitus [21]. The action of this drug is at the skeletal muscle, increasing glucose transport across the cell membrane [22]. Metformin is also known to reduce gene expression of inflammatory mediators such as IL-1 and Monocyte chemoattractant protein (MCP)-1 [23]. Metformin also reduces gene expression of an isoform of cyclophilin, cyclophilin D [24]. It is therefore, tempting to speculate that our observation of reduced plasma cyclophilin A in our patients taking metformin is related to metformin action on cyclophilin A gene expression in monocytes. This hypothesis however needs scrutiny.

In summary, our study reveals that patients with type 2 diabetes have higher circulating levels of the immunophilin cyclophilin A. Our observations that plasma cyclophilin A is higher in patients with type 2 diabetes irrespective of whether they have coronary artery disease or not indicates that hyperglycemia has an effect on cyclophilin secretion. Given that cyclophilin is known to be secreted from monocytes and vascular wall cells in conditions of oxidative stress such as hyperglycemia, the plasma circulating levels of cyclophilin A in patients with diabetes and CAD possibly reflects an increased oxidative stress and proinflammatory status in these conditions. Cyclophilin A thus needs to be evaluated as a marker of proinflammatory status in type 2 diabetes and possibly a predictor of early vascular disease through a prospective study in a large population of diabetic subjects.

## Abbreviations

ANOVA: Analysis of Variance; CAD: Coronary artery disease; CRP: C reactive protein; DM: Diabetes Mellitus; EDTA: Ethylene diamine tetra cetic acid; ELISA: enzyme-linked immunosorbent assay; FBS: fasting blood sugar; GAPDH: Glyceraldehyde 3-phosphate dehydrogenase; HbA1c: Glycated haemoglobin; HDL: High density lipoprotein; LDL: Low density lipoprotein; VSMCs: Vascular smooth muscle cells.

## Competing interests

All authors declare that they have no competing interests.

## Authors’ contributions

SR and CCK designed the study and wrote the paper; AV, VA and DG carried out the experiments; VR, VC, AM, NSP, KRS and MRP planned, executed the study and helped in analyzing the data. All authors read and approved the final manuscript.
